# Detection of the failed-tolerance causes of electronic-portal-imaging-device-based *in vivo* dosimetry using machine learning for volumetric-modulated arc therapy: A feasibility study

**DOI:** 10.1016/j.phro.2025.100785

**Published:** 2025-05-17

**Authors:** Nipon Saiyo, Hironori Kojima, Kimiya Noto, Naoki Isomura, Kosuke Tsukamoto, Shotaro Yamaguchi, Yuto Segawa, Junya Kohigashi, Akihiro Takemura

**Affiliations:** a Department of Quantum Medical Technology, Graduate Course of Medical Sciences and Technology, Division of Health Sciences, Graduate School of Medical Sciences, Kanazawa University, Ishikawa, Japan; b Department of Radiology, Kanazawa University Hospital, Kanazawa University, Ishikawa, Japan; c Faculty of Health Sciences, Institute of Medical, Pharmaceutical and Health Sciences, Kanazawa University, Ishikawa, Japan; d School of Radiological Technology, Faculty of Health Science Technology, Chulabhorn Royal Academy, Bangkok, Thailand

**Keywords:** Failed-tolerance detection, EPID-based *in vivo* dosimetry, Machine-learning model

## Abstract

**Background and Purpose:**

When electronic-portal-imaging-device (EPID)-based *in vivo* dosimetry (IVD) identifies dose tolerance failures, the cause of the failures should be evaluated. This study aimed to develop a machine-learning (ML) model to classify the cause of EPID-based IVD failures in volumetric-modulated arc therapy (VMAT) treatment.

**Materials and Methods:**

Twenty-three prostate VMAT plans were used to recalculate the dose distribution in homogeneous phantom images as no-error (NE) plans. Errors in the randomized multileaf collimator (RMLC) position, monitor unit (MU) variation, lateral position, pitch rotation, and roll rotation were simulated. The IVD results of the NE plans and introduced errors were obtained using EPIgray software. Support vector machines (SVMs) were used to develop ML models for each error. The accuracy percentage, F1-score, and area under the receiver operating characteristic (ROC) curve (AUC) were used to evaluate models’ performances. The models were verified using five additional plans with an Alderson Rando phantom.

**Results:**

The models obtained accuracies of over 90% and F1-scores of 0.9 for the RMLC position and MU variation. For lateral position, pitch rotation, and roll rotation errors, the accuracies were 66.1%, 65.2%, and 66.8%, and the F1-scores were 0.66, 0.65, and 0.67, respectively. The AUCs for all the errors were over 0.7. Additionally, the model verification results consistently classified EPIgray data for all the error types.

**Conclusion:**

The developed ML models classified the causes of the failed tolerance of the EPID-based IVD.

## 1 Introduction

Electronic-portal-imaging-device (EPID)-based *in vivo* dosimetry (IVD) is used to compare the planned dose from the treatment planning system (TPS) with the delivered dose measured by EPIDs in radiation therapy. This method can detect errors related to the delivery device, dose calculation, treatment plan, and patient positioning or anatomy [[1], [2], [3]]. Various software tools exist for IVD analysis, each compatible with different linear accelerators [3].

EPID-based IVD software with the back-projection method has been developed to reconstruct dose distributions using EPID-measured signals on planning computed tomography (CT) images. This system enables comparison between the reconstructed and planned dose distributions at automatically generated points of interest (POIs) within high-dose regions [1,3,4,5]. The result at each POI is typically reported as the percentage dose deviation between the planned and reconstructed doses [[6], [7], [8]]. A 5 % dose deviation threshold at each POI is used as a clinical tolerance based on vendor recommendations [9].

Out-of-tolerance results in EPID-based IVD consistent with issues reported in the American Association of Physicists in Medicine (AAPM) Task Group Report 307 (TG-307) [3,10]. These discrepancies may arise from machine-related errors, treatment planning inaccuracies, anatomical changes, or delivery issues and should be evaluated to determine clinically relevant discrepancies and manage risks in the treatment process. TG-307 recommends evaluating failures by daily setup imaging review, treatment delivery history, and daily quality assurance (QA) results [3,11].

Currently, artificial intelligence (AI) in radiotherapy has been explored for predicting patient-specific QA (PSQA) results (e.g., gamma passing rates) and detecting delivery errors for intensity modulated radiation therapy (IMRT) and volumetric modulated arc therapy (VMAT) techniques [[12], [13], [14], [15], [16], [17], [18], [19], [20], [21], [22], [23]]. Studies also combine AI with transit dosimetry images or fluence maps to identify machine errors, setup errors, and anatomical changes [[24], [25], [26], [27], [28]]. However, to the best of our knowledge, AI applications for detecting tolerance-failure causes in EPID-based IVD using point-dose data have not been reported. Additionally, manual analysis of EPID-based IVD failures is time consuming and prone to human error. The absence of automated tools from EPID-based IVD point-dose data presents a challenge for efficient and accurate QA processes.

Thus, the purpose of this study is to develop a machine-learning (ML) model to classify the causes of failed tolerance in EPID-based IVD for VMAT treatments using point-dose data. It investigates whether ML can accurately identify errors. This is a feasibility study using ML with EPID-based IVD point-dose data from phantom measurements to classify tolerance-failure causes, supporting clinical staff in diagnosing the causes of failed analysis, like an automatic tool for EPID-based IVD processes.

## 2 Materials and methods

### 2.1 EPIgray data

**Fig. 1:**
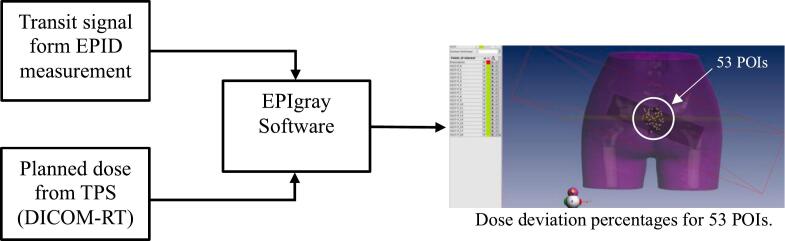
The process for analyzing EPIgray data.

The EPIgray data refers to EPID-based IVD results that serve as input data for the ML model that was analyzed by EPIgray™ software (DOSIsoft, France), comprising dose deviation percentages at 53 POIs (point doses), including the prescribed dose point (PS) and points 0 to 51. In this study, PS refers to point 52. The analyzing process of the EPIgray data is illustrated in Fig. 1. Each point’s dose is calculated using Equation (1) [8,9].

(1) $$\%Dose\;deviation=\frac{\left({\mathrm{Dose}}_{m}-{\mathrm{Dose}}_{\mathrm{TPS}}\right)}{{\mathrm{Dose}}_{\mathrm{TPS}}}\times 100$$

where *Dose_m_* is the dose measured or reconstructed using EPID, and *Dose_TPS_* is the dose calculated from the TPS.

*Dose_m_* at each POI can be calculated as follows:

(2) $${\mathrm{Dose}}_{m}=ISV∙C_{\mathrm{cal}}∙CF∙INV∙\frac{\mathrm{TMR}}{\mathrm{fTMR}}$$

where *ISV* is the image signal at the POIs, *C_cal_* is the dose calibration factor, *CF* is the signal conversion factor for the field size (FS) at the source-to-axis distance (SAD) and the patient’s thickness, *INV* is the geometric distance from the source to the detector or from the source to the POIs, *TMR* is the tissue maximum ratio for the FS at the depth of the isocenter or other POIs, and *fTMR* (finite TMR) is the ratio between two doses measured in the phantom at the EPID level at the depth of the maximum dose (*D_max_*) with and without a finite specific phantom thickness in the beam [3,4,9].

### 2.2 Treatment plan, introduced-error plan, and dose measurement

Twenty-three retrospective VMAT treatment plans for prostate cancer were used to develop measured plans for obtaining EPIgray data, including nineteen plans from RayStation TPS version 10.0 (RaySearch Laboratories AB, Stockholm, Sweden) and four plans from MONACO TPS version 6.1 (Elekta AB, Stockholm, Sweden). All the plans used 6-MV flattening filter-free photon beams, a dose rate of 1,000 monitor units (MU)/min, with one and two arc(s) for 22 and one patient(s), respectively. The measured plans were divided into no-error (NE) and introduced-error plans. Both were delivered via I’mRT phantom (IBA Dosimetry GmbH, Schwarzenbruck, Germany) with an Elekta Infinity linac version 4.0.3 and EPID iViewGT^TM^ version 3.4.1 b519 (Elekta AB, Stockholm, Sweden). Measured data were imported into EPIgray software version 2.0.10 for analyzing EPIgray data, and then the results were exported to develop the ML model.

The 23 treatment plans were transferred to I’mRT phantom images, and the dose distribution was recalculated using MONACO TPS to create the NE plans. The I’mRT phantom was selected for model development because its density aligned with EPIgray’s commissioning data. The introduced errors were focused on those causing tolerance failures in EPID-based IVD, including machine-related and patient-related errors. Machine-related errors were multileaf collimator (MLC) position error and MU variation, while patient-related errors involved patient position errors. This study focused on randomized MLC (RMLC) position errors, which were generated by modifying the MLC positions in 23 prostate cancer plans using MATLAB software v.2024a with a standard deviation (SD) of 0.5 mm. The new MLC movement was both inward and outward at each control point. Modified plans were imported into the MOSAIQ system v.2.64 (Elekta AB, Stockholm, Sweden) for dose delivery to obtain 23 RMLC EPIgray data.

MU variation was performed by adjusting the original MU of the NE plans by ± 5 %, and patient position errors were simulated by shifting the position of the I’mRT phantom from the isocenter on the treatment couch laterally by ± 2 mm and rotating the pitch and roll by ± 2°. These error magnitudes were based on previous studies [23,27,[29], [30], [31]]. Thus, 64 instances of EPIgray data were obtained for each error: MU variation, lateral position (LAT) error, pitch rotation (PIT) error, and roll rotation (ROL) error. Additionally, the accuracy of the EPID-based IVD was ensured before dose measurements, following the vendor’s recommendations, TG-307, and aligned with the recent protocol by Esposito *et al* [3,9,32]*.* This study was approved by the institutional review board (IRB) and ethics committee of Kanazawa University (IRB number 111133–1) on December 13, 2023.

### 2.3 Machine-learning modeling

#### 2.3.1 Training and test datasets

The training and test datasets were composed of EPIgray data from NE and introduced errors. For example, the MU variation model used nineteen NEs, nineteen plus MUs, and nineteen minus MUs for training, while the test dataset included four NEs, four plus MUs, and four minus MUs. At each POI, point-dose data were labeled as P (passing) if they were within a ± 5 % dose deviation or F (failing) if they exceeded ± 5 %. However, POIs missing data were excluded. Prefixes “p” and “m” indicate plus and minus introduced errors, except for the RMLC error, which possesses a single magnitude. NE_P_/NE_F_ represents passing/failing POIs for NE plans. RMLC_P_/RMLC_F_ represent passing/failing POIs for RMLC errors. Additionally, pMU_P_, pLAT_P_, pPIT_P_, pROL_P_, mMU_P_, mLAT_P_, mPIT_P_, and mROL_P_ denote passing POIs for ± 5 % MU, ±2 mm LAT, ±2° PIT, and ± 2° ROL errors, while pMU_F_, pLAT_F_, pPIT_F_, pROL_F_, mMU_F_, mLAT_F_, mPIT_F_, and mROL_F_ denote failing POIs for these errors, respectively. The data sizes and class labels for the RMLC, MU, and position errors are shown in Table 1.

**Table 1:** Training and test datasets and specific class labels related to passed (P) and failed (F) tolerances of EPIgray results.

Model	Magnitude of the error	Number of training data	Number of test data	Specific class
		(Point dose value)	(Point dose value)	Labels
Randomized MLC (RMLC) error	SD 0.5 mm	1,939	460	NE_F_
				NE_P_
		(19 NE and 19 RMLC IVD results)	(4 NE and 4 RMLC IVD results)	RMLC_F_ RMLC_P_
MU variation	±5% of MU	2,914	714	NE_F_
				NE_P_
		(19 NE and 38 MU variation IVD results)	(4 NE and 8 MU variation IVD results)	mMU_F_ mMU_P_ pMU_F_ pMU_P_
Lateral position (LAT) error	±2 mm	2,911	766	NE_F_
				NE_P_
		(19 NE and 38 Lateral position shift IVD results)	4 NE and 8 Lateral position IVD results)	mLAT_F_ mLAT_P_ pLAT_F_ pLAT_P_
Pitch rotation (PIT) error	±2°	2,809	767	NE_F_
				NE_P_
		(19 NE and 38 pitch rotation IVD results)	4 NE and 8 pitch rotation IVD results)	mPIT_F_ mPIT_P_ pPIT_F_ pPIT_P_
Roll rotation (ROL) error	±2°	2,863	766	NE_F_
				NE_P_
		(19 NE and 38 Roll rotation IVD results)	(4 NE and 8 roll rotation IVD results)	mROL_F_ mROL_P_ pROL_F_ pROL_P_

For example, the training dataset for the LAT error included 2,911-point doses from 53 POIs, comprising 19 NE, 19 plus LAT, and 19 minus LAT error IVD results. The test dataset included 766-point doses from 4 NE, 4 plus LAT, and 4 minus LAT error IVD results. POIs missing data were excluded.

#### 2.3.2 Model development

**Fig. 2:**
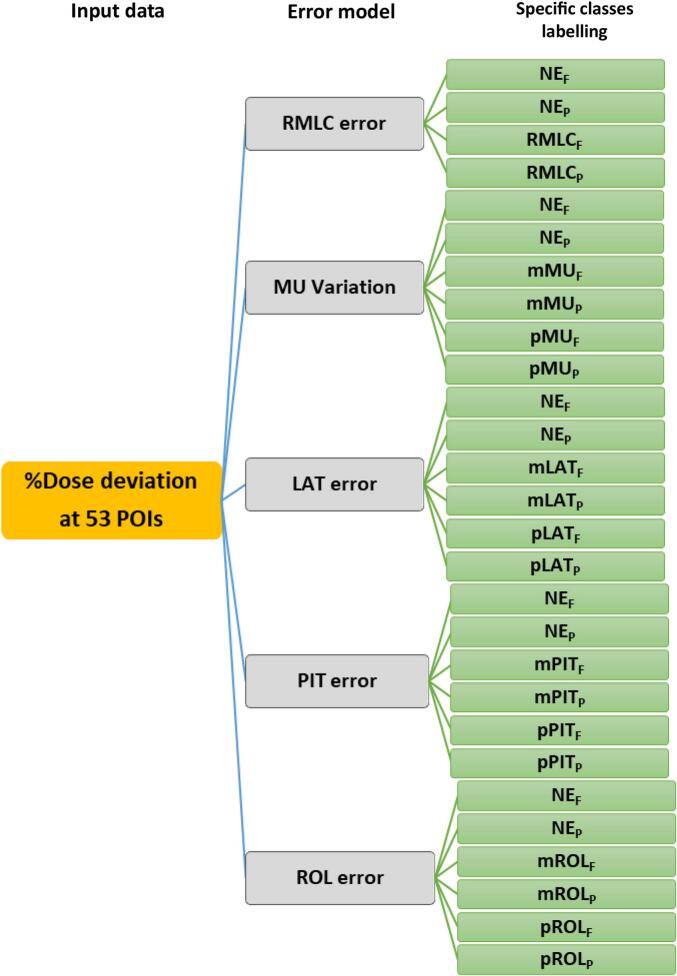
Workflow of the development of the five ML models to identify the causes of failed tolerance based on EPIgray results.

The model was developed using support vector machines (SVMs) with multiclass classification of all the error types (four classes for RMLC and six classes for the other errors) with polynomial and Gaussian kernels. Training was conducted using five-fold cross-validation in MATLAB (Statistical and Machine-Learning Toolbox version 2024a). Five error models were developed to classify the RMLC position, MU variation, LAT, PIT, and ROL errors. The workflow of the model development is illustrated in Fig. 2. The model’s performance was assessed using the accuracy percentage, F1-score, and area under the receiver operating characteristic (ROC) curve (AUC). Because of the class imbalance, the F1-score was selected as the evaluation metric for multiclass classification. The accuracy percentage and F1-score were computed using Equations (3), (4), respectively. However, multiple ML algorithms were initially evaluated using multiclass classifications, including SVMs, decision trees, ensemble methods, and neural networks with EPIgray data and five-fold cross-validation. SVMs showed the best performance based on the accuracy percentages, F1-scores, and AUCs and were, therefore, selected. The detailed performances of all the tested models are provided in the supplementary material.

(3) $$\%Accuracy=\frac{\mathrm{TP}+TN}{\mathrm{TP}+FP+FN+TN}\times 100$$

(4) $$F1-score=2\times \frac{\mathrm{Precision}\times Recall}{\mathrm{Precision}+Recall}$$

where TP is the number of true positives, where the model correctly predicts a POI as class X (e.g., RMLC_F_); TN is the number of true negatives, where the model correctly predicts a POI as not class X; FP is the number of false positives, where the model incorrectly predicts a POI as class X; FN is the number of false negatives, where the model incorrectly predicts a POI as not class X; precision is the fraction of correct positive predictions; and recall is the fraction of correctly identified positive instances.

For example, for the RMLC error model possessing four classes, if class X is RMLC_F_, TP means that the model correctly predicted RMLC_F_; TN means that the model correctly predicted not RMLC_F_; FP means that the model incorrectly predicted RMLC_F_; and FN means that the model incorrectly predicted not RMLC_F_. For MU, LAT, PIT, and ROL error models possessing six classes, the TP, TN, FP, and FN definitions are similarly applied. The model predictions from all 53 POIs were used to calculate accuracy percentages and F1-scores by the microaverage method, and AUCs were reported for each class to evaluate the model’s discriminative capability across all the classes.

### 2.4 Model verification

The models were verified to study the feasibility of implementing them on different geometric data, such as patients, using five VMAT prostate cancer treatment plans that were not included in the training or test datasets. The dose distributions of the five plans were recalculated using MONACO TPS on Alderson Rando pelvis phantom images (RAN-110, Phantom Laboratory). The errors were introduced as described above, including MU variation and position errors, while the RMLC position error was generated by modifying the MLC positions of the five phantom plans with a 0.5 mm SD in MATLAB and then reimporting the modified plans into MOSAIQ for dose delivery. Measurements were conducted using EPID on the Alderson Rando pelvis phantom, and the results were analyzed with EPIgray software and exported to the models. Then, the accuracy percentages and microaverage F1-scores were calculated for each case and error model.

## 3 Results

### 3.1 Model performance

The AUCs for the RMLC position error and MU variation classification were over 0.9 for each error class. This demonstrates the ability of the SVM model to detect RMLC position errors and MU variation based on EPIgray data. However, the AUCs for all the position errors of the passing tolerance data with introduced plus and minus errors were more than 0.7, and the AUCs for the NE plans with both passing and failing tolerances and for the failing tolerance result of the introduced plus and minus errors were more than 0.9. These results show sufficiently good performance to classify the cause of the tolerance failure for all the position errors. The results of the five error models are shown in Table 2.

**Table 2:** Performances of the five tolerance-failure detection models in the training and test datasets.

Tolerance-failure detection model	Accuracy (%)		F1-score	Specific Classes	AUC values of ROC curve
	Training data	Test data	Test data	Test data	Test data
RMLC position error	99.6	99.3	0.99	NE_F_	1.000
				NE_P_	0.996
				RMLC_F_	1.000
				RMLC_P_	1.000
MU variation	90.9	91.2	0.91	NE_F_	0.989
				NE_P_	1.000
				mMU_F_	0.996
				mMU_P_	0.963
				pMU_F_	1.000
				pMU_P_	0.952
LAT error	67.8	66.1	0.66	NE_F_	1.000
				NE_P_	1.000
				mLAT_F_	0.987
				mLAT_P_	0.775
				pLAT_F_	0.979
				pLAT_P_	0.778
PIT error	67.9	65.2	0.64	NE_F_	1.000
				NE_P_	1.000
				mPIT_F_	0.923
				mPIT_P_	0.754
				pPIT_F_	0.916
				pPIT_P_	0.757
ROL error	67.3	66.8	0.67	NE_F_	0.999
				NE_P_	1.000
				mROL_F_	0.985
				mROL_P_	0.787
				pROL_F_	0.986
				pROL_P_	0.790

### 3.2 Model verification

The verification results show that the models can detect all the causes of the failed tolerance of the IVD results in different phantom geometric data with five VMAT plans. These verification results are detailed in Table 3.

**Table 3:** Model verification results for the five tolerance-failure detection models.

Tolerance-failure detection model	Accuracy (%)	F1-score
RMLC position error	100.0 ± 0.0	1.00 ± 0.00
MU variation	95.3 ± 6.1	0.95 ± 6.10
LAT error	66.0 ± 2.3	0.66 ± 0.02
PIT error	66.8 ± 0.7	0.67 ± 0.01
ROL error	68.1 ± 1.8	0.68 ± 0.02

## 4 Discussion

The study results demonstrate the potential of ML for detecting causes of failed tolerance in EPID-based IVD results for VMAT treatment using EPIgray data (point doses). SVM multiclass classification was used to develop models because it effectively handles nonlinear and imbalanced data, especially EPIgray data, outperforming other algorithms in terms of the AUCs for all the classes, as shown in the supplementary data. To approximate our clinical scenario, we focused on the RMLC position, MU variation, and translational and rotational position errors as the main sources of treatment error during delivery. The RMLC position error model achieved high classification accuracy, and the AUCs in the test dataset were above 0.9 for all the classes. In addition to its highly accurate verifications for different phantom geometries, the RMLC position error model also achieved a high average accuracy (100 ± 0.00 %) and a high average F1-score (1.00 ± 0.00), indicating the model’s highly consistent classification of the failed tolerance of the EPIgray data because of the RMLC position error, even when evaluated using new geometric data. To the best of our knowledge, although no previous study has been conducted using SVMs and EPIgray data, previous research on SVMs using other PSQA input data to detect MLC position errors can be compared with this work. Nyflot *et al.* used linear SVM classification to detect the presence or absence of MLC position error with the radiomic features of gamma images for IMRT PSQA. They simulated a 2-mm shift for systematic and randomized MLC errors*.* Their study used binary classification (NE and any MLC error) and a multiclass classification (NE, systematic error, and random error), and they found that the accuracy of the binary classification (77.3 %) was higher than that of the multiclass classification (64.3 %) [14]. By contrast, our model achieved high accuracy (99.30 %) in the test dataset with multiclass classification of the RMLC error, with an SD of 0.5 mm. The differences in the input data and SVM function produce different accuracies, and our study illustrates the potential of SVMs with polynomial and Gaussian functions for classifying EPIgray point dose results. Furthermore, Sakai *et al.* used SVMs and the radiomic features of the gamma maps measured using EPID in the PSQA of IMRT for detecting MLC positional errors of 0.5 and 1.0 mm. Their results showed high sensitivity (1.000), high specificity (0.818), and large AUCs (0.998) similar to our results [23]. Nonetheless, their study showed the ability of SVM binary classification with EPID non-transit data in PSQA to detect RMLC errors, whereas we used SVM multiclass classification with EPID transit dosimetry data. Our results indicate the ability of SVM multiclass classification to detect RMLC positional errors using EPIgray data.

The MU variation model showed a strong ability to classify the results out of tolerance of the EPIgray data, and its AUCs in the test dataset were over 0.9 for all error classes. Moreover, the verification results were close to the model’s performance for both the accuracy and F1-score, and the results also showed highly consistent classification for different geometric data. Thus, the model can detect the cause of the failed tolerance of EPIgray data from ± 5 % of the MU. However, to the best of our knowledge, no previous reports on SVMs for detecting MU variation are available in the literature. Therefore, the results of other algorithms and input data were compared. Huang *et al.* implemented a random forest algorithm with multiclass classification to identify MU variations of ± 1, ±3, and ± 5 % using the image-based features of EPID for PSQA, and found that the accuracy was 100.0 % for MU variation [30], whereas our result was 91.2 % for ± 5 % of the MU. This is similar to the results of their study, even for differences in the input data and algorithm. Thus, our model can classify the cause of failed tolerance caused by MU variation using EPIgray data.

All the position error models showed moderate accuracies of 66.1 %, 65.2 %, and 66.8 % and F1-scores of 0.66, 0.64, and 0.67 for LAT, PIT, and ROL errors, respectively. However, a previous study by Zhang *et al.* used SVMs with the radiomic features of EPID images to detect positioning errors. They simulated a range of translational position errors, including left to right (LR), superior to inferior (SI), and anterior to posterior (AP), errors of 0, 2, and 4 mm. Moreover, they expect that the model can detect errors exceeding 3 mm. Their F1-scores were 0.758, 0.793, and 0.741, and their AUCs were 0.71, 0.91, and 0.78 for LR, SI, and AP errors, respectively [26]. Our F1 scores for the LAT error, which is the LR error in Zhang *et al.*'s study, were lower than theirs because our study employed a six-class classification for position error models, making it more complex than their binary classification approach. Additionally, our AUCs were above 0.7 for all the classes related to their study, and the verification results obtained for different geometric data were consistent. Consequently, our model classifies the lateral shift-induced failed tolerances of EPIgray IVD result.

Nevertheless, to the best of our knowledge, PIT and ROL errors have not been classified using SVMs in previous studies. Our work demonstrates SVMs’ ability to classify failed tolerance in EPIgray data because of rotational errors. All the AUCs exceeded 0.7 across all the classes, and model verifications using different phantom data were consistent. Thus, SVMs effectively classify the causes of failed tolerance in EPIgray data according to lateral shifts of ± 2 mm and pitch/roll rotational shifts of ± 2°.

In conclusion, this study demonstrated the development of SVM classification model combined with EPIgray data (point-dose numerical data) to detect the causes of failed tolerance in EPIgray data. The models classified the five causes of failed tolerance in EPID-based IVD results for VMAT treatment. Our approach can extend to other EPID-based IVD platforms if the input data have similar characteristics. However, it may not be applicable to transmission-based systems that rely on image-based data. Moreover, this study is limited by the use of data from a single treatment site and phantom measurements, considering only five causes of tolerance failures. However, applying this model for real-patient data remains challenging due to their greater complexity compared to phantom-based data. Therefore, future studies should explore additional treatment sites, real patient geometric data, varying error magnitudes, and other potential causes to enhance the model’s accuracy and efficiency.

## CRediT authorship contribution statement

The authors confirm their contribution to the paper as follows.

**Study conception and design:** Nipon Saiyo, Hironori Kojima, and Akihiro Takemura.

**Treatment planning, equipment preparation, and data collection:** Nipon Saiyo, Hironori Kojima, Kimiya Noto, Naoki Isomura, Kosuke Tsukamoto, Shotaro Yamaguchi, Yuto Segawa, Junya Kohigashi, and Akihiro Takemura.

**Model development**, **data analysis and interpretation of results:** Nipon Saiyo and Akihiro Takemura

**Draft manuscript preparation:** Nipon Saiyo and Akihiro Takemura

**Final approval of the version to be published:** Nipon Saiyo, Hironori Kojima, Kimiya Noto, Naoki Isomura, Kosuke Tsukamoto, and Akihiro Takemura

All authors reviewed the results and approved the final version of the manuscript.

## Declaration of competing interest

The authors declare that they have no known competing financial interests or personal relationships that could have appeared to influence the work reported in this paper.

## References

[1] Mijnheer B. (2019). EPIDs and QA of advanced treatments. J Phys Conf Ser.

[2] McCurdy B.M.C. (2023). EPID-based in vivo dosimetry–new developments and applications. J Phys Conf Ser.

[3] Dogan N., Mijnheer B.J., Padgett K., Nalichowski A., Wu C., Nyflot M.J. (2023). AAPM Task Group Report 307: Use of EPIDs for Patient-Specific IMRT and VMAT QA. Med Phys.

[4] Francois P., Boissard P., Berger L., Mazal A. (2011). In vivo dose verification from back projection of a transit dose measurement on the central axis of photon beams. Phys Med.

[5] Olaciregui-Ruiz I., Beddar S., Greer P., Jornet N., McCurdy B., Paiva-Fonseca G. (2020). In vivo dosimetry in external beam photon radiotherapy: requirements and future directions for research, development, and clinical practice. Phys Imaging Radiat Oncol.

[6] Rajasekar A., Moggré A., Cousins A., Marsh S. (2020). Optimising the use of EPIgray for 3DCRT breast treatments. Phys Eng Sci Med.

[7] Halliday S.D., Day R.A., Greig L., Louwe R.J. (2022). Clinical pilot study for EPID-based in vivo dosimetry using EPIgray™ for head and neck VMAT. Phys Eng Sci Med.

[8] Puente S.W., Gallego M.C., Martínez D.S., Fernández R.C., Fuentes J.D.G., Suárez A.B.C. (2025). Working thresholds for in-vivo dosimetry in EPIGray based on a clinical, anatomically-stratified study. Phys Med.

[9] Dosisoft. EPIgray Expert Guide. Version 2.0.10. Cachan, France: Dosisoft; 2021.

[10] Nailon W.H., Welsh D., McDonald K., Burns D., Forsyth J., Cooke G. (2019). EPID-based in vivo dosimetry using Dosimetry Check™: overview and clinical experience in a 5-yr study including breast, lung, prostate, and head and neck cancer patients. J Appl Clin Med Phys.

[11] Bossuyt E., Weytjens R., Nevens D., De Vos S., Verellen D. (2020). Evaluation of automated pre-treatment and transit in-vivo dosimetry in radiotherapy using empirically determined parameters. Phys Imaging Radiat Oncol.

[12] Chan M.F., Witztum A., Valdes G. (2020). Integration of AI and machine learning in radiotherapy QA. Front Artif Intell.

[13] Osman A.F., Maalej N.M. (2021). Applications of machine and deep learning to patient-specific IMRT/VMAT quality assurance. J Appl Clin Med Phys.

[14] Nyflot M.J., Thammasorn P., Wootton L.S., Ford E.C., Chaovalitwongse W.A. (2019). Deep learning for patient-specific quality assurance: Identifying errors in radiotherapy delivery by radiomic analysis of gamma images with convolutional neural networks. Med Phys.

[15] Ono T, Iramina H, Hirashima H, Adachi T, Nakamura M, Mizowaki T. Applications of artificial intelligence for machine-and patient-specific quality assurance in radiation therapy: current status and future directions. J Radiat Res 2024;rr ae033. doi:10.1093/jrr/rrae033. 10.1093/jrr/rrae033 PMC11262865 38798135

[16] Wall P.D., Fontenot J.D. (2020). Application and comparison of machine learning models for predicting quality assurance outcomes in radiation therapy treatment planning. Inform Med Unlocked.

[17] Kimura Y., Kadoya N., Tomori S., Oku Y., Jingu K. (2020). Error detection using a convolutional neural network with dose difference maps in patient-specific quality assurance for volumetric modulated arc therapy. Phys Med.

[18] Kimura Y., Kadoya N., Oku Y., Kajikawa T., Tomori S., Jingu K. (2021). Error detection model developed using a multi-task convolutional neural network in patient-specific quality assurance for volumetric-modulated arc therapy. Med Phys.

[19] Sivabhaskar S., Li R., Roy A., Kirby N., Fakhreddine M., Papanikolaou N. (2022). Machine learning models to predict the delivered positions of Elekta multileaf collimator leaves for volumetric modulated arc therapy. J Appl Clin Med Phys.

[20] Yoganathan S.A., Ahmed S., Paloor S., Torfeh T., Aouadi S., Al-Hammadi N. (2023). Virtual pretreatment patient-specific quality assurance of volumetric modulated arc therapy using deep learning. Med Phys.

[21] Bosco L.D., Franceries X., Romain B., Smekens F., Husson F., Le Lann M.V. (2023). A convolutional neural network model for EPID-based non-transit dosimetry. J Appl Clin Med Phys.

[22] Li G., Duan L., Xie L., Hu T., Wei W., Bai L. (2024). Deep learning for patient-specific quality assurance of volumetric modulated arc therapy: prediction accuracy and cost-sensitive classification performance. Phys Med.

[23] Sakai M., Nakano H., Kawahara D., Tanabe S., Takizawa T., Narita A. (2021). Detecting MLC modeling errors using radiomics-based machine learning in patient-specific QA with an EPID for intensity-modulated radiation therapy. Med Phys.

[24] Wolfs C.J., Canters R.A., Verhaegen F. (2020). Identification of treatment error types for lung cancer patients using convolutional neural networks and EPID dosimetry. Radiother Oncol.

[25] Bedford J.L., Hanson I.M. (2022). A recurrent neural network for rapid detection of delivery errors during real-time portal dosimetry. Phys Imaging Radiat Oncol.

[26] Zhang X., Dai G., Zhong R., Zhou L., Xiao Q., Wang X. (2021). Radiomics analysis of EPID measurements for patient positioning error detection in thyroid associated ophthalmopathy radiotherapy. Phys Med.

[27] Zeng Y., Li H., Chang Y., Han Y., Liu H., Pang B. (2024). In vivo EPID-based daily treatment error identification for volumetric-modulated arc therapy in head and neck cancers with a hierarchical convolutional neural network: a feasibility study. Phys Eng Sci Med.

[28] Olaciregui-Ruiz I., Simões R., Jan-Jakob S. (2024). Deep learning-based tools to distinguish plan-specific from generic deviations in EPID-based in vivo dosimetry. Med Phys.

[29] Mijnheer B., Jomehzadeh A., González P., Olaciregui-Ruiz I., Rozendaal R., Shokrani P. (2018). Error detection during VMAT delivery using EPID-based 3D transit dosimetry. Phys Med.

[30] Huang Y., Pi Y., Ma K., Miao X., Fu S., Chen H. (2023). Image-based features in machine learning to identify delivery errors and predict error magnitude for patient-specific IMRT quality assurance. Strahlenther Onkol.

[31] O'Daniel J., Hernandez V., Clark C., Esposito M., Lehmann J., McNiven A. (2025). Which failures do patient‐specific quality assurance systems need to catch?. Med Phys.

[32] Esposito M., Baldoni R., Bossuyt E., Bresciani S., Clark C.H., Jones M. (2024). A commissioning protocol for portal imaging-based radiotherapy in vivo dosimetry systems. Phys Imaging Radiat Oncol.

