# Supplementary material for: Detection of the failed-tolerance causes of electronic-portal-imaging-device-based in vivo dosimetry using machine learning for volumetric-modulated arc therapy: A feasibility study
Source: Phys Imaging Radiat Oncol. 2025 May 17;34:100785. doi: 10.1016/j.phro.2025.100785 (PMC12148416; doi:10.1016/j.phro.2025.100785)
Supplement: Supplementary Data 1 [file mmc1.pdf]

Detection of the failed-tolerance causes of electronic-portal-imaging-device-based *in vivo* dosimetry using machine learning for volumetric-modulated arc therapy: A feasibility study

Supplementary Materials

Table S1
The model performance with 5-folds cross-validation for multi-algorithms of five failed tolerance error models with Classification Learner in MATLAB software for RMLC error model.

Classification algorithm	%Accuracy		F1-score on Test data set	Specific classes	AUC values On Test data set
	Training data	Test data			
Decision tree	99.9	99.8	0.99	NE_F	0.9898
				NE_P	0.9995
				$RMLC_F$	0.9997
				$RMLC_P$	0.9995
SVM (Polynomial)	99.6	99.3	0.99	NE_F	1.0000
				NE_P	0.9960
				$RMLC_F$	1.0000
				$RMLC_P$	1.0000
Ensemble Bagged Trees	99.9	99.6	0.99	NE_F	0.9998
				NE_P	0.9996
				$RMLC_F$	0.9999
				$RMLC_P$	0.9999
Logistic Regression	96.2	89.8	0.90	NE_F	0.6180
				NE_P	0.9334
				$RMLC_F$	0.6817
				$RMLC_P$	0.9230
Neural Network	91.9	92.0	0.92	NE_F	0.9680
				NE_P	0.9999
				$RMLC_F$	0.9999
				$RMLC_P$	0.9605

Table S2
The model performance with 5-folds cross-validation for multi-algorithms of five failed tolerance error models with Classification Learner in MATLAB software for MU variation model.

Classification algorithm	%Accuracy		F1-score on Test data set	Specific classes	AUC values On Test data set
	Training data	Test data			
Decision tree	89.6	91.3	0.91	NE_F	0.9993
				NE_P	0.9959
				mMU_F	0.9976
				mMU_P	0.9536
				pMU_F	0.9888
				pMU_P	0.9432
SVM (Gaussian)	90.9	91.2	0.91	NE_F	0.9894
				NE_P	1.0000
				mMU_F	0.9962
				mMU_P	0.9637
				pMU_F	0.9997
				pMU_P	0.9519
Ensemble Bagged Trees	86.7	91.9	0.91	NE_F	0.9998
				NE_P	0.9998
				mMU_F	0.9999
				mMU_P	0.9587
				pMU_F	0.9997
				pMU_P	0.9025
Logistic Regression	80.1	80.3	0.80	NE_F	0.7578
				NE_P	0.9687
				mMU_F	0.9994
				mMU_P	0.9715
				pMU_F	0.9935
				pMU_P	0.9573
Neural Network	91.9	92.0	0.92	NE_F	0.9680
				NE_P	0.9999
				mMU_F	0.9999
				mMU_P	0.9605
				pMU_F	0.9994
				pMU_P	0.9466

Table S3

The model performance with 5-folds cross-validation for multi-algorithms of five failed tolerance error models with Classification Learner in MATLAB software for LAT error model.

Classification algorithm	%Accuracy		F1-score on Test data set	Specific classes	AUC values On Test data set
	Training data	Test data			
Decision tree	67.1	64.49	0.64	NE_F	0.3984
				NE_P	0.9859
				$mLAT_F$	0.9856
				$mLAT_P$	0.7837
				$pLAT_F$	0.4809
				$pLAT_P$	0.7885
SVM (Polynomial)	67.8	66.1	0.66	NE_F	1.0000
				NE_P	1.0000
				$mLAT_F$	0.9874
				$mLAT_P$	0.7754
				$pLAT_F$	0.9785
				$pLAT_P$	0.7779
Ensemble Bagged Trees	64.4	66.3	0.66	NE_F	1.0000
				NE_P	0.9996
				$mLAT_F$	0.9868
				$mLAT_P$	0.7760
				$pLAT_F$	0.4623
				$pLAT_P$	0.7687
Logistic Regression	64.1	60.7	0.60	NE_F	0.7748
				NE_P	0.9718
				$mLAT_F$	0.4851
				$mLAT_P$	0.7183
				$pLAT_F$	0.4829
				$pLAT_P$	0.7949
Neural Network	67.1	66.4	0.67	NE_F	1.0000
				NE_P	0.9960
				$mLAT_F$	0.9666
				$mLAT_P$	0.7755
				$pLAT_F$	0.4765
				$pLAT_P$	0.7854

Table S4
The model performance with 5-folds cross-validation for multi-algorithms of five failed tolerance error models with Classification Learner in MATLAB software for PIT error model.

Classification algorithm	%Accuracy		F1-score on Test data set	Specific classes	AUC values On Test data set
	Training data	Test data			
Decision Tree	66.8	63.2	0.63	NE_F	0.9993
				NE_P	0.9960
				$mPIT_F$	0.6694
				$mPIT_P$	0.7411
				$pPIT_F$	0.6874
				$pPIT_P$	0.7470
SVM (Gaussian)	67.9	65.2	0.66	NE_F	1.0000
				NE_P	1.0000
				$mPIT_F$	0.9225
				$mPIT_P$	0.7539
				$pPIT_F$	0.9158
				$pPIT_P$	0.7565
Ensemble Bagged Trees	67.5	66.0	0.66	NE_F	0.9990
				NE_P	0.9998
				$mPIT_F$	0.8763
				$mPIT_P$	0.7651
				$pPIT_F$	0.7708
				$pPIT_P$	0.7659
Logistic Regression	65.4	58.9	0.64	NE_F	0.7752
				NE_P	0.9718
				$mPIT_F$	0.6500
				$mPIT_P$	0.6864
				$pPIT_F$	0.3449
				$pPIT_P$	0.7167
Neural Network	68.4	61.7	0.62	NE_F	1.0000
				NE_P	0.9960
				$mPIT_F$	0.9905
				$mPIT_P$	0.7217
				$pPIT_F$	0.6958
				$pPIT_P$	0.7234

Table S5
The model performance with 5-folds cross-validation for multi-algorithms of five failed tolerance error models with Classification Learner in MATLAB software for Roll error model.

Classification algorithm	%Accuracy Training data	Test data	F1-score on Test data set	Specific classes	AUC values On Test data set
Decision Tree	67.5	64.2	0.64	NE_F	0.9993
				NE_P	0.9958
				$mROL_F$	0.7005
				$mROL_P$	0.7439
				$pROL_F$	0.7031
				$pROL_P$	0.7448
SVM (Gaussian)	67.3	66.8	0.67	NE_F	1.0000
				NE_P	1.0000
				$mROL_F$	0.9225
				$mROL_P$	0.7539
				$pROL_F$	0.9158
				$pROL_P$	0.7565
Ensemble Bagged Trees	65.0	65.8	0.66	NE_F	0.9999
				NE_P	0.9999
				$mROL_F$	0.9539
				$mROL_P$	0.7493
				$pROL_F$	0.9447
				$pROL_P$	0.7555
Logistic Regression	64.3	61.4	0.61	NE_F	0.7749
				NE_P	0.9718
				$mROL_F$	0.4282
				$mROL_P$	0.7438
				$pROL_F$	0.7867
				$pROL_P$	0.6786
Neural Network	66.6	66.6	0.67	NE_F	0.9930
				NE_P	0.9834
				$mROL_F$	0.8710
				$mROL_P$	0.7786
				$pROL_F$	0.9077
				$pROL_P$	0.7810
